# Croatian Society of Radiology (1928–2008), the Croatian Medical Association – 80 years of existence and activity

**DOI:** 10.2478/v10019-011-0003-x

**Published:** 2011-03-15

**Authors:** Slavko Simunic, Kresimir Glavina, Nada Besenski, Ratimira Klaric-Custovic

**Affiliations:** 1 Department of Diagnostic and Interventional Radiology, University Clinical Centre Osijek, Osijek, Croatia; 2 Department of Diagnostic and Interventional Radiology, University Clinical Centre Split, Split, Croatia; 3 Department of Diagnostic and Interventional Radiology, University Hospital “Sestre milosrdnice“, Zagreb, Croatia

**Keywords:** radiology, history, Croatian Medical Association, Croatian Society of Radiology

## Abstract

Often and in various connotations one can hear or read the following syntagma: “Let’s leave the past in the past - and turn to the future”. Even more frequent and numerous are opposite opinions, *e.g*. “There is no future without past”, “Future is built on past” or “Remembering our past – reaching for our future”, and many more.

## The first practical use of X-rays in medicine

In the very same year (1895) as Francis Joseph I, the Austro-Hungarian emperor, ceremonially opened a new building of the Croatian National Theatre in Zagreb, Wilhelm Konrad Röntgen, a German physicist (Lennep 1845 – Munich 1923), in his experimental laboratory in Würzburg discovered the radiation until then unknown, which he called X-rays. As a proof then, he also published the first roentgenogram – an image of his wife’s hand with a ring on the middle finger. He was awarded the first Nobel Prize in physics (1901) for this extraordinary discovery, and the newly discovered rays were named the Roentgen rays in his honour. The discovery gave origin to a new profession (science) radiology (roentgenology), which enabled a fresh impetus in the development not only of medicine, but of many other human activities.

The first practical use of X-rays in medicine in this region was recorded in Rijeka (1897), when a roentgen apparatus was acquired, Prof. Dr. Peter Salcher presented at the Naval Academy an X-ray of a hand with a ring on the baroness Vranyczány’s finger. Thereafter followed Ogulin, Šibenik and Srijemska Mitrovica (1898), Zagreb (1901), Osijek (1902) and Lepoglava (1904) – let us remember that the Paulists of Lepoglava, in rivalry with the Jesuits, started providing university education (1656) with lectures in logic. The roentgen apparatuses were then obtained in Pula, Split, Dubrovnik, Bjelovar (1905), and Varaždin, Karlovac, Vinkovci and Nova Gradiška (1911), and Sisak (1912). We may also mention that the roentgen radiation was for the first time used for the research in palaeontology by Prof. Dragutin Gorjanović-Kramberger (1902), a Croatian natural scientist of world-renowned, a geologist, palaeontologist and anthropologist, for taking X-ray of the Krapina early man’s jaw.^1^

## Organizations in Croatia

Organised medical work in Croatia started with the establishment of the **Društvo slavonskih liječnika** (*Slavonian Medical Association)* in Osijek (1874), that started publishing the **Glas slavonskih liječnika** (*Slavonian Medical Journal*) (1877) – the today’s “Medicinski vjesnik” (*Medical Journal),* published by the University Hospital Osijek, the Faculty of Medicine in Osijek, and other regional medical centres. A few months later, in the same year (1874), the **Sabor liečnika kraljevine Hrvatske i Slavonije** (*Medical Association of the Kingdom of Croatia and Slavonia)* was founded in Zagreb, and started publishing the **Liečnički viestnik** (*Medical Journal)* (1877) – the today’s “Liječnički vjesnik” (*Medical Journal of the Croatian Medical Association)*, the gazette of the Croatian Medical Association.^1^ From the 1920s, the Association was called the **Hrvatski** **liječnički zbor** (Croatian Medical Association), and from 1945 to 1992 it operated under the name the **Zbor liječnika Hrvatske** (*Medical Association of Croatia)*, becoming the member of the Savez lekarskih/liječničkih društava Jugoslavije (*Union of Medical Associations of Yugoslavia)*.

As the aggression on Croatia started (1991), the Medical Association of Croatia freezed (on 26^th^ February 1991), and subsequently broke off all the relations (on 30^th^ September 1991) with the Union of Medical Associations of Yugoslavia. We were also obligated to do so by the UN Security Council Resolution no. 757/1992, stating that “....*any activity in the area of science, technology, information sciences, education, culture and sports, even publishing activity, with the SFRY /Socialist Federative Republic of Yugoslavia/ shall be suspended*...”. In that period, and observing the then-valid and customary schedule of rotating the seats of the leadership of a particular professional association during a 4-year mandate, Zagreb was the seat of the Yugoslav Society of Cardiology, the Yugoslav Association of Pulmonology and Phthisiology, the Association of Yugoslav Clinical Cytology, the Yugoslav Association of Anaesthesiologists, the Yugoslav Association of Dermatology, and the Yugoslav Society of Radiology and Nuclear Medicine. All the presidents, vice-presidents and secretaries of these associations resigned their duties, and on behalf of their respective associations renounced any further cooperation. The **Section of radiology** of the Medical Association of Croatia, in accordance with this, broke off (on 4^th^ October 1991) the cooperation with the Yugoslav Society of Radiology and Nuclear Medicine, and returned the mandate for presiding the Society for the period 1988 – 1992, and renounced to the obligation of organising the 14^th^ Congress of the Yugoslav Society of Radiology and Nuclear Medicine planned for 1992, informing about it all the professional associations of the then Yugoslav republics and provinces.

Following the disintegration of the Socialist Federative Republic of Yugoslavia, and the founding of the Republic of Croatia, with its international recognition, the conditions were met for the direct membership of Croatian professional associations in international professional institutions, so that the Croatian Medical Association also became a full member of the **World Medical Association** (1992), *i.e.* the **Croatian Medical Association**.

Although the radiology profession and science in this region was being applied soon after the respective discovery (Rijeka 1897), the roentgenologists / radiologists were formally organised only in 1928, through the foundation of the **Society of Roentgenology** (1928–1935), which changed the name into the **Section of Radiology, Electrophysiology and Balneology** (1935–1945). After the Second World War, the Society was reorganised again and the name changed to the **Section of Radiology and Nuclear Medicine** (1945–1984), having radiologists, radiotherapeutists, oncologists and nuclear medicine practitioners as members. The development of nuclear medicine and the growing number of educated nuclear medicine practitioners lead to the separation of these experts into two independent sections, so that ours changed the name into the **Section of Radiology** of the Medical Association of Croatia (1984 – 1992).

As the Croatian Medical Association became a member of the World Medical Association, all the Sections were entitled to a higher level of membership, *i.e.* to receive the title of a society, so that on this basis the present-day **Hrvatsko društvo radiology (HDR) – The Croatian Society of Radiology (CSR)** was founded and named at the Founding Assembly held on 22^nd^ April 1992, in the Great Hall of the Croatian Medical Association, being the successor of all the already mentioned Associations of Radiology in Croatia.

Since the foundation (1928) until the present day, the Sections / Societies were headed by presidents: Prof. Dr. Laza Popović (1928–1945), Prof. Dr. Ferdo Petrovčić (1945–1958), Prof. Dr. Vladimir Gvozdanović (1958–1960), Prof. Dr. Silvije Kadrnka (1960–1965), Ass. Prof. Dr. Sc. Ivo Belančić (1965–1967), Prim. Dr. Karlo Strohal (1967–1971), Prof. Dr. Sc. Šime Čičin-Šain (1971–1976), Prof. Dr. Sc. Duško Katunarić (1976–1986), Prof. Dr. Sc. Davorin Kovačević (1986–1992), Prof. Dr. Sc. Slavko Šimunić (1992–1996), Prof. Dr. Sc. Nada Bešenski (1996– 2004), Prof. Dr. Sc. Ratimira Klarić-Čustović (2004– 2008), and Prof. Dr. Sc. Boris Brkljačić (from 2008 on).

Immediately after its foundation, the CSR obtained right to apply for an independent direct membership in international institutions. Firstly, all the required documentation for the membership in the **European Association of Radiology - EAR** was collected and submitted (a written application and an explanation in English, an English version of the Statute of the Croatian Medical Association and the Rules of Procedure of the Society, and the list of the Society’s members). The application was examined in the General Assembly of the European Association of Radiology at the time of the European Congress of Radiology, in Vienna 1993. The application was granted unanimously, and the CSR became a regular and full member of the EAR.

An application for the membership of the **International Society of Radiology – ISR** followed. In accordance with the same procedure, the application waited for the General Assembly and the International Congress of Radiology to be held – in Singapore 1994, when the CSR became a member of this society as well.

## Professional activities

The Croatian radiologists have always had a significant impact on the organization and participation in various radiology events in the then Yugoslavia: the 1^st^ Yugoslav Meeting on Radiology (Split, 1930), organised by Prim. Dr. Jakša Račić; the 2^nd^ Yugoslav Meeting on Radiology (Belgrade, 1935), in the organisation participated the following colleagues from Croatia: Prof. Dr. Ernst Mayerhofer – the dean of the Faculty of Medicine / Zagreb, Prof. Dr. Laza Popović, radiologist / Zagreb, Dr. Milan Smokvina, radiologist / Zagreb, and Dr. Stevo Radojević, radiologist / Zagreb. After the Second World War, meetings were held every four years, by rotation in the republics’ capitals. The 1^st^ Scientific Meeting of Radiologist of the Federal People’s Republic of Yugoslavia (FNRY) – (Belgrade 1950); the 2^nd^ Scientific Meeting of Radiologists of FNRY (Zagreb 1953). Thereafter, the meetings were named congresses: the 3^rd^ Congress of Radiology of FNRY (Ljubljana 1956); the 4^th^ Congress of Radiology of FNRY (Skopje 1960); the 5^th^ Congress of Radiology of Yugoslavia (Belgrade 1964). Because of the two Scientific Meetings held earlier (Split 1930, and Belgrade 1935), the next congress was titled the 8^th^ Congress of Radiology of Yugoslavia (Pula 1968); and the 9^th^ Congress of Radiology of Yugoslavia (Ljubljana 1972). Out of the total of 228 lectures held on that congress, 55 lectures (24 %) were from Croatia. At the 10^th^ Congress of Radiology of Yugoslavia (Sarajevo 1976), out of the total of 245 lectures held, 65 lectures (26%) were from Croatia. At the 11^th^ Congress of Radiology of Yugoslavia (Novi Sad 1980), out of the total of 396 lectures held, 97 lectures (25%) were from Croatia. At the 12^th^ Congress of Radiology of Yugoslavia (Belgrade 1984), out of the total of 325 lectures held, 97 lectures (20%) were from Croatia. And at the 13^th^ Congress of Radiology of Yugoslavia (Ohrid 1988), out of the total of 496 lectures held, 97 lectures (20%) were from Croatia. The 14^th^ Congress of Radiology of Yugoslavia (1992) was supposed to be held in organisation of the Croatian radiologists, but due to the known war situation, Croatia renounced the task.

**FIGURE 1.**
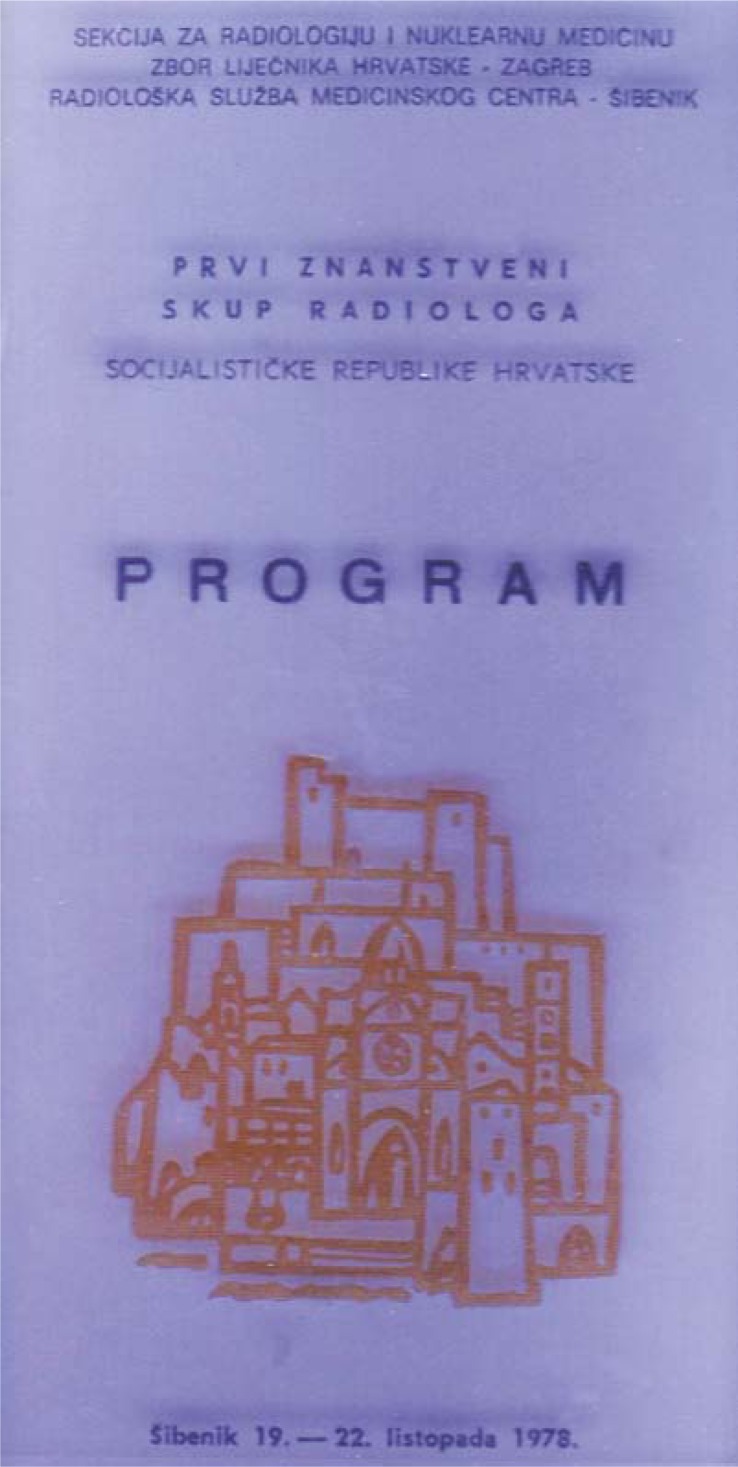
Programme of the First scientific meeting of radiologists of the Croatia, Šibenik, October 19–22, 1978.

In the period from 1978 to 1992, and owing to Prof. Dr. Sc. Duško Katunarić and Prim Dr. Krešo Pavleković, the president and the secretary respectively of the then Section of Radiology, the Section organised **Scientifics Meetings of Radiologists of Croatia** in various towns in Croatia, followed an alternating “continent – seaside” territorial principle. Even though in those times the meetings were of mere republic-importance, they had wide reverberation across the entire then Yugoslavia, both among the participants and the lecturers, so those meetings were considered to be at the “level” of a congress. There were ten meetings held in total: in Šibenik (1978) (Figure 1), Plitvička jezera (1979), Split (1981), Osijek (1982), Pula (1985), Karlovac (1986), Opatija (1987), Požega (1989), Zadar (1990) and Varaždin (1992). The total of 776 lectures were held at the meetings (752 national authors and 24 foreign authors – 5 from Germany, 5 from Switzerland, 2 from Italy, 2 from Norway, 2 from the USA, 1 from France, 1 from Belgium, 1 from Sweden), and 102 lectures at three Courses (on Radiology of Kidney, on Radiology of Mediastinum, and on CT in Neuroradiology).

With the development of radiology and an increasing number of specialists in radiology, as well as their professional and scientific orientation, the conditions were met, as per the Statute of the Croatian Medical Association and the Rules of Procedure of the Croatian Society of Radiology, for the establishment of particular sections. Firstly, the **Section of Neuroradiology** (1933) was founded – the first president was Prof. Dr. Sc Nada Bešenski. Then followed the establishment of the **Junior Radiologists Forum** (1994) – encouraged by the Junior Radiologist Forum (JRF) of the European Association of Radiology – the first president was Dr. Franka Jelavić – Kojić. The third to be established was the **Section of Ultrasound in Medicine** (1994) – the first president was Prof. Dr. Sc. Ivo Drinković. Then the **Section of Interventional Radiology** (2000) – the first president was Prof. Dr. Sc. Josip Mašković, and the **Section of Thoracic Radiology** (2001) – the first president was Prof. Dr. Sc. Zlata Herceg – Ivanovi.

On several occasions the Section / the Society took part in specialised programmes of the international fair “Medicine and Technology” at the Zagreb Fair. The *Symposium on Interventional Radiology* (1981) – chaired by S. Šimunić; the *Symposium on Percutaneous Transluminal Angioplasty – PTA* (1983) – chaired by S. Šimunić / M. Šesto, (a book was published: “*PTA renalnih, perifernih i koronarnih* arterija. Šimunić S, Šesto M, editors. Zagreb; 1985”); the *Symposium on Percutaneous Organ and Organic Systems Drainage* (1985) – organized / chaired by S. Šimunić / I. Obrez – Ljubljana; the *Symposium on MRI in Clinical Medicine* (1988) – chaired by S. Šimunić / D. Ivančević; the *Symposium on Rationalization of Diagnostic Procedures in Radiology, Nuclear Medicine and Ultrasound* (1987) – chaired by S. Šimunić / S. Franić; the *Symposium on Algorithm of Diagnostic Procedures in Neuroradiology* (1999) – chaired by S. Šimunić / N. Bešenski; the *Symposium on Teleradiology* (1999) – chaired by A. Hebrang / S. Šimunić and assoc.

The emergence of therapeutic procedures in interventional radiology and our first experiences were presented at the CSR expert meeting: the *Round Table on Interventional Radiology* (1980) – chaired by S. Šimunić, also published in a book: “*Okrugli stol o intervencijskoj radiologiji*. Šimunić S, Gürtl R, editors, 1981”.

The CSR has had an intensive, long-term cooperation with members of other professions and institutions. It has endeavoured to approach and solve a number of common problems with the related Radiation Protection Association. It used to cooperate, at the time, with radiologists of the Department of Roentgenology and Physical Therapy of the Faculty of Veterinary Medicine in Zagreb (Prof. Dr. Sc. Mensur Šehić). With the Ministry of Health and Social Welfare of the Republic of Croatia and the Croatian Institute for Health Insurance, the CSR discussed various professional, status and organisational issues on the regular basis. Expert meetings are well-attended and held regularly (9–10 times per year), in cooperation with other clinical professions (internal medicine specialists, paediatricians, neurosurgeons, urologists, orthopaedists, otorhinolaryngologists, etc.)

Various business partners, manufacturers and suppliers of equipment, accessories and expandable supplies presented to us regular basis novelties in their assortment of products: Siemens, General Electric, Philips, Shimadzu, Bayer Health Care, Schering, Farma, Sonimed, Thomy Frey East, Mark/De Plano, Medtronic, OptiMed, Bard, Abbot, Bracco, Cook and many others.

A long-term professional, loyal and friendly cooperation with the Hungarian Radiological Society (“Societatis Radiologorum Hungarorum”) was particularly emphasized, dating back since 1985, when the *Vereinbarung über die Ungarischen und Jugoslawischen Radiologischen Gesellschaften* was signed (signatories: *Gy Vargha and B. Fornet* *– Budapest; M. Radojević – Skopje; S. Šimunić – Zagreb and L. Popović – Novi Sad.* At the same year, during the International Fair “Medicine and Technology”, at the Zagreb Fair, within the frame-work of professional events at the Symposium on Percutaneous Organ and Organic Systems Drainage, the lectures were given by Gy Vargha and T. Baranyai (Debrecen), L. Horváth (Péch) and Lélek (Zalaegerszeg). The cooperation was continued through regular joint Croatian-Hungarian Radiological Symposiums: Kőszeg (1999) and Opatija (2000), after which Slovenia also joined this cooperation, so the symposia were thereafter held under the name Hungarian-Croatian-Slovenian Radiological Symposia: Pécs (2001), Maribor (2002), Koprivnica (2003), Héviz (2004), Maribor (2005). It was at that point decided that the symposia were to be held biannually from then: Vukovar (2007) and Kehidakustanyi (2009). The participants of the meeting in Vukovar visited and paid respects by placing wreaths at the Stone Cross, on the confluence of Vuka and Danube, and at the Memorial Cemetery of the Homeland War victims on Ovčara.^2^

The CSR gave an appreciation award to the Hungarian Radiological Society for the close long-term cooperation, and the same award was also given to the following individuals: Gy Vargha, G. Vadon, J. Kénez, B. Fornet, and L. Hórvath at the congress in Tihany, in 1996. N. Bešenski was awarded a certificate - Honorary Member of the Hungarian Society of Neuroradiology (7th Annual Meeting of the Hungarian Society of Neuroradiology - Györ 1997).

The following colleagues were declared honorary members of the Societatis Radiologorum Hungarorum: N. Bešenski (Zagreb), K. Glavina (Osijek), S. Šimunić (Zagreb / Osijek), I. Lovasić (Rijeka) and B. Brkljačić (Zagreb).

It has become customary to participate in one another’s national, i.e. Croatian and Hungarian, radiology congresses: in Miskolc 1994, Opatija 1994; Tihany 1996, Osijek 1998^3^, Pécs 1998, Split 2002^4^, etc., but also in the Hungarian Society of Neuroradiology congresses.

**FIGURE 2.**
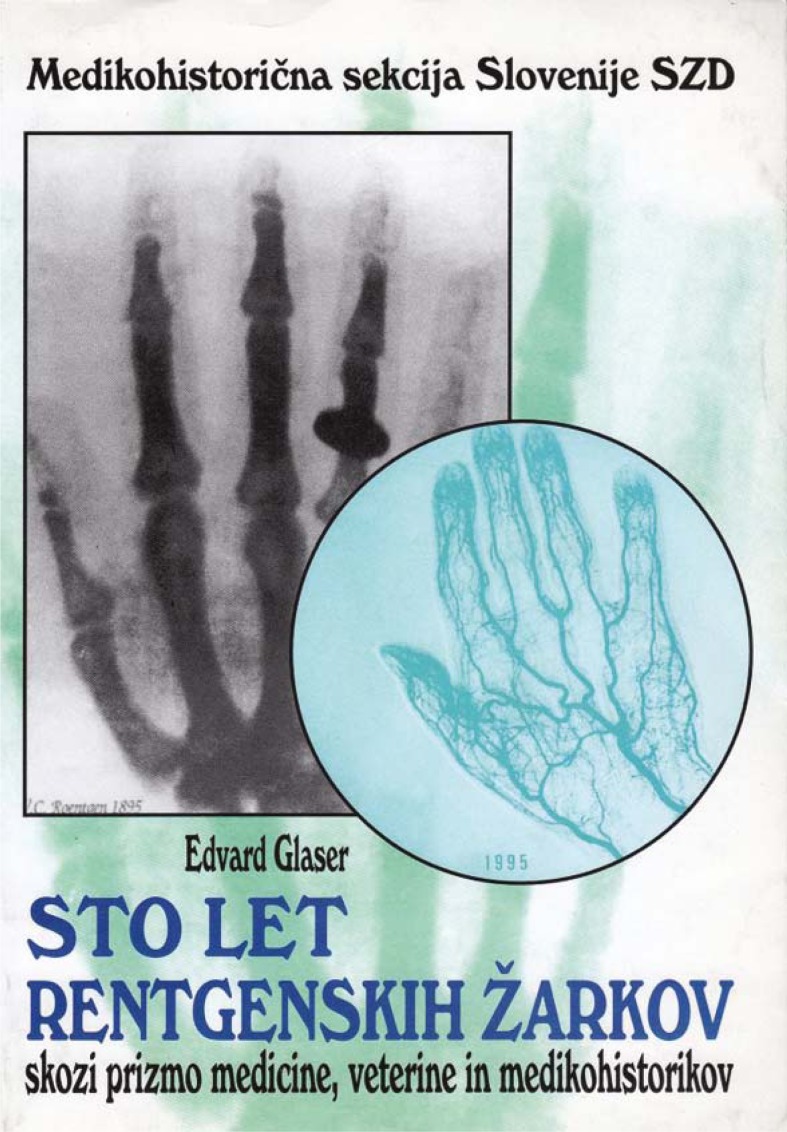
The book of Edvard Glaser. *Sto let rentgenskih žarko kozi prizmom medicine, veterine in medikohistorikov [100 years of the x-rays through the prism medicine, veterinary nad medical historyans]*. Glaser E, editor. Maribor: Medikohistorična sekcija Slovenije, SZD [Section for the history of medicine, Slovenian Medical Society]; 1998.^5^

The cooperation with the Združenje radiologov Slovenije (the Slovenian Association of Radiology) went back long ago. There used to be held (1958–1971) joint meetings of Croatian and Slovenian radiologists. The last one, the sixth expert meeting, was held in Zagreb, in 1971. At that point, the cooperation was developed on Croatian and Slovenian national radiology congresses: in Portorož 1996, Portorož 2000, Ljubljana 2004, Ptuj 2008. Moreover, the Croatian radiologists cooperated occasionally on expert meetings with the University Medical Center Ljubljana and the Maribor General Hospital / University Medical Centre Maribor. On the occasion of the 100^th^ anniversary of the Röntgen’s discovery, the Medicohistorical Section of the Slovenian Medical Association published a book including an article by Croatian authors: “Lovasić I, Šimunić S, Borković Z, Pavan G. *Prve rentgenske snimke i prvi rentgen aparati u Hrvatskoj*. (*The first roentgen rays and roentgen apparatuses in Croatia).* Maribor; 1995” (Figure 2). The international radiology cooperation to be mentioned is the participation of the Croatian delegation at the Founding Conference of the Radiological Society of Bosnia and Herzegovina (Sarajevo 1996). The Society was after that admitted to the European Association of Radiology (1999). The Croatian radiologists participated in congresses of the Radiological Society of the Medical Association of Bosnia and Herzegovina (Sarajevo 1999, Tuzla 2003, Sarajevo 2007).

The Croatian radiologists participated almost on the regular basis with lectures in congresses of the European Association of Radiology (ECR) in Vienna, as well as in the International Congress of Radiology (ICR). They, for example, held five lectures and presented two posters in the Vienna ECR ’93, while young radiologists, members of the CSR’s Junior Radiologists Forum, won three Winners of the Day medals.

We have been participating already for decades in expert meetings of the ALPE-ADRIA bordering countries, Austria, Italy, Slovenia and Croatia, held in various towns. The examples of our participation are the following: “Gvozdanović V, Nutrizio V, Šimunić S. La nostra esperienza con Emi Scanner. Padova; 1975”, and “Gvozdanović V, Nutrizio V, Šimunić S, Marinšek Čičin-Šain V. CT in the Diagnostic Acousticus Neurinoma”.

There are meetings of the Cardiovascular and Interventional Radiological Society of Europe (CIRSE), whose members are: Z. Čačić, A. Hebrang, J. Mašković, S. Šimunić, L. Camby-Sapunar, V. Vidjak, V. Tkalec, held every second year in various European towns, with the participation of a dozen or more Croatian radiologists.

Our teachers and the then authorities on radiology (S. Kadrnka, M. Smokvina, V. Gvozdanović, D. Katunarić, M. Bašić), owing to their personal acquaintances and friendships, to relations and cooperation with leading European and other authorities on radiology, organised in those times the following participation and lectures: “Jirout J. Pneumomyelography of the Cervical Spine. Prague; 1965”; “Vieten H. Die Methoden der Kontrastmitteldarstellung des Herzens und der großen Gefäße deren Indikationen und Gefahren. Düsseldorf ; 1967”; “Wellauer J. Kontrastmittel Probleme in der modernen Röntgendiagnostik. Zurich; 1967”; Bodar P. La radiologie du grêle (maladie de Crohn-Tuberculose-Tumeurs“). Louvain, Belgium; 1968”; Oliva L. Studio radiologico dell’incontinenza urinaria femminile. Siena; 1968”.

In more recent times, and through the offices of D. Kovačević, M. Lovrenčić, N. Bešenski, S. Šimunić, V. Vidjak, B. Brkljačić and others, we were hosts to S. Wallace (Houston/USA), Pocajt (Maribor), D. Pavčnik (Ljubljana), H. Hricak and A. Margulis (San Francisco/USA), J. Matela (Maribor), L. Hórvath, C. Focafy, M. Kovey (Pécs/Hungary); Ufflacker (Charleston/USA), and others.

The CSR and its members were entrusted the organisation of the “Workshop – New Application of CT and MRI“ – Elscint, Zagreb, 1996; “Visiting Junior Radiologists to Eastern Europe“, Zagreb, 1995, 1997; a “Crash Course in CT”, on the occasion of the acquisition of a large number of CT devices by the Ministry of Health of the Republic of Croatia, and the Croatian Institute for Health Insurance.

It is worth remembering, recording and saving from oblivion the works on radiology, from the old days, but also from the most recent times, written by radiology experts and teachers. Among them, the book of Hodges *et al* was the first comprehensive teaching material and great help to students in acquiring necessary knowledge, in addition to lectures, seminars and exercises for the Radiology course (Table 1).

In addition to the publications listed in Table 1, the CSR members radiologists published countless number of articles and chapters in journals, encyclopaedias, and books, both in Croatia and around the world, those being not only in the field of radiology but also in other professions: Marotti M, Klarić-Čustović R, Lovrenčić M, Krolo I, Papa J, Agbaba M, Radanović B, Mandić A, Štern-Padovan R, Mašković J, Sučić Z, Čavka K, Glavina K, Borković Z, Ivanovi-Herceg Z, Camby-Sapunar L, Brkljačić B, Kalousek M, Brajša M, Drinković I, Jakovac I, Gürtl R, Klenkar M, Čičin-Šain Š, Marinšek- Čičin-Šain V, Sabolić A, Prpić-Hartl V, Bešenski N., S. Šimunić and many more.

The then Section of Radiology, in cooperation with the central health institutions (the then University Hospital “Dr. M. Stojanović” in Zagreb, and the University Hospital Centre Zagreb), organized memorial meetings: a **Memorial Meeting Dedicated to the 5^th^ Anniversary of he Death of Prof. dr. Silvije Kadrnka (1902–1965)** – in 1970, and an **In Memoriam Symposium “Vladimir** **Gvozdanović”** (1914–1979) – in 1987.

A **Celebration of the 65^th^** **Anniversary of the Croatian Society of Radiology (1928–1993)** was held, with art programme and a performance by the Physicians Singers of Zagreb choir, and a historical overview in presence of numerous members of the CSR and the invitees. On that occasion, the first logo of the CSR was also presented, designed by Krešimir Ivanček, academic painter – graphic artist, Studio Color Soft, of Bjelovar, and after our ideas and efforts made by Prim. Dr. Luka Ježek, the Head of the Department of Radiology in the Bjelovar General Hospital.

**FIGURE 5.**
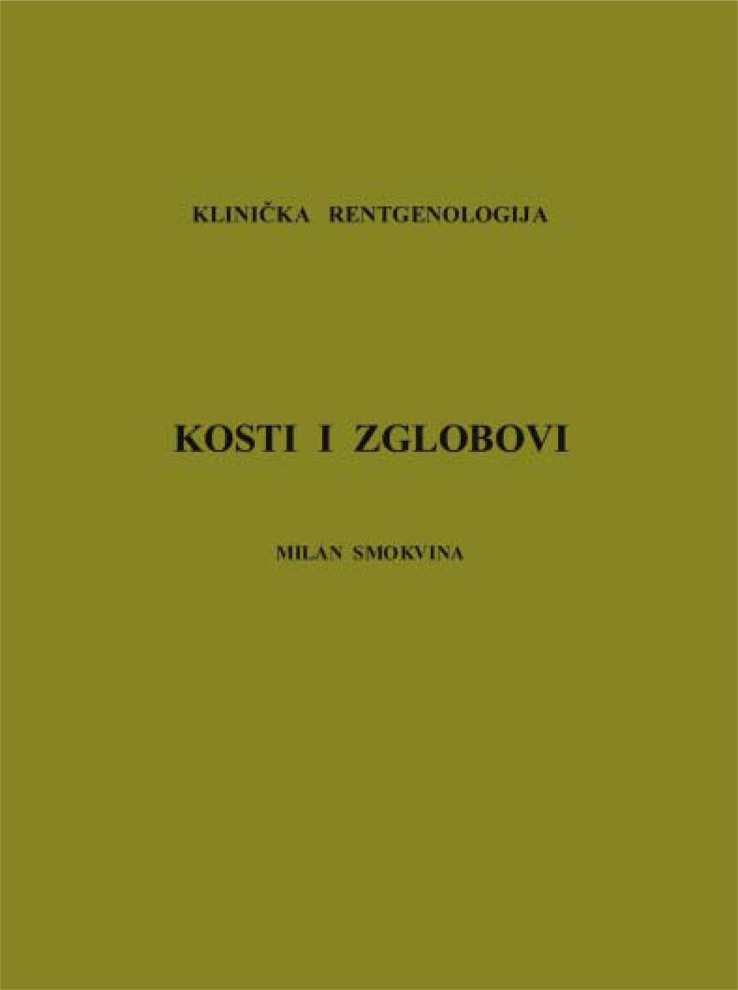
The book of Milan Smokvina. Clinical roentgenology, bones and joints. Smokvina M, editor. Zagreb: Yugoslav Academy of Sciences and Arts; 1959.

There was also held a **Celebration of the 100^th^** **Anniversary of the X-rays Discovery (1985–1995)**. The Society thus joined marking of that event, remembered all around the world. On that occasion the Technical Museum Zagreb organised an exhibition on the **Röntgen Rays Discovery 1985–1995** (Figure 5); and an article: “Lovrenčić M, Marotti M, Bašić M. *Iz povijesti medicinske primjene rentgenskih* *zraka u dijagnostičke svrhe u Hrvatskoj (From the history* *of medical application of roentgen rays for diagnostic purposes in Croatia)”* was published, among others, in the accompanying book.^11^

## Professional journals

Before the Second World War, a journal **Radiološki glasnik** *(Journal of Radiology)* used to be published for some time. The journal **Radiologia Iugoslavica** used to be published from 1966–1992, being a gazette of the Yugoslav Society of Radiology and Nuclear Medicine. Out of 16 members of the Colegium Redactorum until 1974, there were seven (44%) members from Croatia: Bašić M, Gvozdanović V, Mark B, Martinčić N, Petrovčić F, Smokvina M and Špoljar M. Just as an example of the participation of Croatian radiologists in the journal, let us mention that out of the total of 9 articles in one particular issue (*Radiol Yugosl* 8(1); 1974) 4 of them (44%) were from Croatia. In the period from 1974–1990, out of the total of 27 Editorial Board members, 5 of them (18 %) were from Croatia: Ivančević D, Lovrenčić M, Popović S, Spaventi Š, Leković A, and out of the total of 24 Advisory Board members, 5 of them (21%) were from Croatia: Kovačević D, Šimonović I, Šimunić S, Dujmović M, Lovasić I. Just as an example of the participation of Croatian radiologists in the journal, let us mention that out of the total of 27 articles in one particular issue (24[4];1990), 10 of them (37%) were from Croatia.

A joint journal has been published since 1992 – **Radiology and Oncology**, a journal devoted to the publication of original contributions in diagnostic and interventional radiology, CT, US, MR, nuclear medicine, radiotherapy, clinical and experimental oncology, radiobiology, radiophysics and radiation protection. The founders and publishers are the Slovenian Association of Radiology, the Slovenian Nuclear Medicine Society, the Slovenian Society for Radiotherapy and Oncology and the Slovenian Cancer Society, and the **Croatian Medical Association / Croatian Society of Radiology**. The associated societies are also the Societas Radiologorum Hungarorum and the Friuli-Venezia Giulia regional group of La Società italiana per la radiologia medica (SIRM). The first editor-in-chief for many years was Tomaž Benulič (now editor-in-chief-emeritus). He was succeeded by Gregor Serša. Out of the 38 Editorial Board members (1992–2008), 9 of them (24%) were from Croatia: Bešenski N, Boko H, Drinković I, Hebrang A, Lovrenčić M, Osmak M, Papa J, Šimunić S, Lovasić I. The other members were from Slovenia, Hungary, Austria, Italy, USA, Australia, the Netherlands, Canada and Germany. Since year 2008, out of the total of 30 Editorial Board members, 3 of them (10%) are from Croatia: Osmak M, Štern-Padovan R, Miletić D. The journal is published 4 times per year, only in the English language.^12^

## Current activities and congresses

Regular monthly expert meetings (8–10 times per year) are held as a rule in the Great Hall of the Croatian Medical Association, in Šubićeva Street in Zagreb. These meetings used to be held occasionally also in institutions out of Zagreb: in Bjelovar, Sisak, Karlovac, Varaždin, Krapinske toplice and even in Osijek, Split and Pula.

Since the independence of Croatia and the founding of the Croatian Society of Radiology, five Elective Assemblies were held: 1992, 1996, 2000, 2004 and 2008. Thus, the conditions were met (64 years from its foundation and continuous activities, and 95 years from the first practical application of X-rays in Croatia) for holding national radiology congresses.

**The First Congress of the Croatian Society of Radiology with International Participation** was held in Opatija, from 11^th^ – 15^th^ October 1994, in the Adriatic Hotel. On behalf of the European Association of Radiology, the Congress was greeted by the then president, Lodovico dalla Palma (Trieste), and other invitees and guests. Out of the total of 155 lectures and 14 workshops, there were sixteen lectures (10%) and seven workshops (50%) from abroad. Beside the Croatian and foreign expert lectures, part of the lectures was dedicated also to historic topics on the development of radiology in the region: Šimunić S. (part of Croatia); Ježek L. (Bjelovar), Kačić P. (Dubrovnik) and Lovasić I. (Rijeka). There was a book also published on that occasion: “*Dijagnostička i intervencijska radiologija* *(Diagnostic and interventional radiology).* Lovasić I, Dujmović M, Budiselić B, Riman S, editors.^13^

**The Second Congress of the Croatian Society of Radiology with International Participation** was held in Osijek, from 23^rd^ – 25^th^ April 1998, in the Croatian Army Hall.^3^ Out of the total of 150 lectures and 38 posters, there were 20 lectures (13%) and 13 posters (34%) from abroad. On the same occasion a book by Frković M was presented: *“Radiološki atlas probavnog sustava djece (A radiological atlas of the children digestive track)”,* and also a satellite mini-symposium was held*: “Nonionic Radiological Diagnostic Contrast Medium – OPTIRAY (Ioversol)”,* by the company Byk-Gulden, Konstanz, Germany. In addition to the expert topics, the Congress also discussed historic issues related to radiology in the region: Glavina K, Vugrinec M, Dlouhy B, Sontacchi B. The second section of lectures was dedicated to current organisational developments in radiology and radiation protection legislation (Hebrang A, Grgić S, Kubelka D, Vekić B). The proceedings were also published, including lectures and summaries.

**The Third Congress of the Croatian Society of Radiology with International Participation** was held in Split, from 5^th^ – 8^th^ June 2002, in the Marjan Hotel. Out of the total of 176 lectures and 80 posters, there were 53 lectures (30%) and 13 posters (16%) from abroad. On the occasion of the congress, a special issue of Acta Clinica Croatica (*Acta Clin Croat* 2002; 41[Suppl 1]: 1–126.) was published, which included 19 congress articles.^4^

**The Fourth Congress of the Croatian Society of Radiology with International Participation** was held in Zagreb, from 11^th^ – 14^th^ October 2006, in the Westin Hotel. Out of the total of 177 lectures and 51 posters, there were 34 lectures (19%) and 3 posters (6%) from abroad. The medical journal published a special issue (*Lijec Vjesn* 2006; 128[Suppl 7]: 5–107.) with summaries of lectures and posters. (12)

## Key note lectures

The topics of expert meetings and other events in radiology were various and the intention was to follow all the new events. It is evident from the archive material that various techniques were discussed as early as in 1964: lympho-gamma-scintigraphy (Spaventi Š, Bosnar M); lymphography technique with oil contrast medium (Temer B, Beronić I), roentgen cinematography (Katunarić D), on the principles of logetron work (Katunarić D.), on rotation and pendulum roentgen therapy (Bašić M), and a Report on the 1^st^ Symposium on Radiology Protection (Petrovčić F), held in Portorož, was submitted.

A number of interesting radiology cases from daily practice (Hočušćak I) were discussed at the meeting held in the Varaždin General Hospital in 1965, and a lecture was given with reference to the World Congress of Radiology in Rome (Katunarić D).

More than 40 years ago (1967) the application of ultrasound in diagnosis had been discussed (Ašperger Z).

Lectures on radiology were not seldom programmed together with other clinical professions, as *e.g.* a lecture on “Three Cases of Urological Diagnostics” (Kos V. and assoc. radiologists and Čečuk LJ and assoc. urologists – 1968). Or cooperation between internal medicine-radiology for the lecture: “Brachial angiography” (Čustović F, Gvozdanović V, Šimunić S – 1968); “Unusual form of congenital lymphoedema” (cooperation of radiology-surgery Prpić-Hartl V. Pasini M – 1968); “A case of morbus Klippel-Trenaunay-Weber” (Lovrenčić M, Georgijević A – 1968).

Particularly interesting lectures were given by clinicians from other branches before radiological audience: “Coordinated findings of radiologists and clinicians in pulmology“ (internal medicine specialist Harambašić H – 1968); “Biliary tract diseases and role of roentgen diagnostics as viewed through the prism of clinicians” (internal medicine specialist Čerlek S – 1968); “Clinical aspects of chronic gastritis” (internal medicine specialist Kallai L – 1968).

A lecture on “Lumbar myelography without anaesthesia using Mallinckrodt contrast media Conray 60” was held on an expert meeting in Pula (1969).

On pneumogynecography technique and application: lectured by Gjurin B – 1969; on transhepatic cholangiography: Temer B – 1969; on testicular lymphography: Agbaba M and assoc. – 1969.

On the occasion of a visit paid by the colleagues from Ljubljana, two reports were submitted from the 12^th^ International Congress of Radiology – Tokyo 1969, Tabor L from Ljubljana and Gvozdanović V from Zagreb.

In a whole-evening expert programme on “bronchitis”, the topic was discussed by pulmonologists and radiologists from the aspect of the clinical picture, the problems of public health service, the role of functional analysis of breathing and roentgen diagnostics – 1970).

In a review of the 2^nd^ Diagnostic Course of the International Society of Lymphology (Davos 1971), Mihajlović N, Šimunić S, and Agbaba M talked about the organisation of the course, the lymphogram analysis, new nomenclature and new views in lymphography diagnostics.

## Cooperations

In working and cooperating with many companies, manufacturers and suppliers of the necessary working material, there are occasionally held joined meetings aimed at providing information on new products, technologies and methods. A round table on enteroclysis was held (1992): “The small barium enema (enteroclysis) with barium and methylcellulose“ and “Die Technik der radiologischen Dündarmdiagnostik – Enteroclysis“, with companies Nicholas GmbH Sulzbach and Aspro-Nicholas Vienna, with the participation of lecturers: Antes G (Sulzbach) and Holacky (Vienna), and the radiologists: Mandić A, Dolenčić P, Frković M, Kapetanović D and Tonković V, and the internal medicine specialists: Papa B, Vucelić B and Bilić A of Zagreb.

Another round table was held (1992) on “Ultrasound in algorithm of diagnostic and interventional procedures in renal disease”. Introductory lectures were held by Odak D: “Ultrasound diagnostics in inflammatory and obstructive renal diseases”; Drinković I: “Ultrasound diagnostics of expansive renal processes and intervention ultrasound”; and Brkljačić B: “Possibilities of application of Doppler and colour-Doppler in renal diseases”. Sabljar-Matovinović M, Mrklić B, Agbaba M, Hebrang A, Marotti M and Kunštek N participated in the discussion.

The CSR occasionally cooperates with the Slovenian colleagues – radiologists. Pavčnik D (1993), of the Institute of Diagnostic and Interventional Radiology, the University Clinical Center Ljubljana, filled the whole-evening programme with lectures on intravascular stents, microcatheters, subselective angiographies, transcatether embolizations and fibrinolysis and vena cava filters.

An ever-topical issue of the protection of patients and personnel from ionizing radiation was covered in two whole-evening programmes (1993) by the most competent experts in the field: Hebrang A and Gunarić M – the Ministry of Health of the Republic of Croatia; Milković-Kraus S, Cerovac H and Cesar D – the Institute for Medical Research and Occupational Health; Pokupec R – the Department of Ophthalmology, University Hospital Centre Zagreb; Vekić B – the Ruđer Bošković Institute; and Marović F – the Ministry of Labour and Social Welfare of the Republic of Croatia.

The CSR contributed also to the celebration on the occasion of the 150^th^ anniversary of the Bjelovar General Hospital and Radiology Activities (1995), when the lectures were, beside the hosts (Ježek L and assoc.), held by Marotti M and assoc; and Papa J and assoc.

The CSR and the Radiological Diagnostics Department of the Karlovac General Hospital organised a joint expert meeting (1995), with the programme prepared by the hosts, which covered the historic development of radiology in Karlovac (Pavan G); algorithm of radiological examinations in abdominal tumour diagnostics (Pavan G and assoc.); ten years of the ultrasound application in Karlovac (Popović A and assoc.); the value of ultra-sound in diagnostics of choledocholithiasis (Baškot A and assoc.).

Along with one of the expert programmes (1995), an occasional Christmas meeting was held, with a lecture on the comparison between CT and DSA in evaluating the degree of the extension of bronchial carcinoma (Marušić P and assoc.), and an occasional Christmas address was given by Msgr. Matija Stepinec, the parish priest of St. Peter’s Parish in Zagreb; it was followed by a “Little Christmas Concert” (Snježana Arbanas, soprano, medical student ABD, accompanied by piano played by Hlavomirka Bledšnajder, BAMus).

In one of the meetings (1996), the Ministry of Health informed the CSR’s members about the condition of the radiological equipment in the Republic of Croatia, in comparison with other countries (Hebrang A); about the ionizing radiation legislation in force in relation to world standards (Grgić S and assoc.), and about the results of the checks performed on medical sources of radiation (Tonković V).

The cooperation with colleagues radiologists – veterinary surgeons has already been pointed, but let us mention also that the CSR (1966) took part in the celebration on the occasion of the 65^th^ anniversary of the foundation and work of the Department of Roentgenology and Physical Therapy of the Faculty of Veterinary Medicine in Zagreb, when an overview of the historical development of the Department, and scientific and professional cooperation with human medicine were presented (Šehić M).

The CSR and the Croatian Radiation Protection Association (1966) held a joint meeting, with two particularly attractive, interesting and unusual lectures: “Control without destruction in industry” (Krstelj V, Dean of the Faculty of Mechanical Engineering and Naval Architecture in Zagreb), and “Non-destructive methods in research and protection of works of art” (Braun M, Director of the Institute for the Conservation of Objects of Art, Zagreb).

The Society members had an opportunity to listen to and experience another interesting and unusual programme when the then Section of Radiology was led by the president (1976–1986) Prof. Duško Katunarić (1923–1986), and when Tomislav Ladan (1932–2008), a Croatian writer, linguist, translator, polyglot and lexicographer, held a lecture titled: “Literature and medicine”.

This overview of the 80 years of work is a mere attempt to remember, burdened with the present, the “good old times” and save from oblivion at least one part of our rich radiological heritage. We are well aware of many shortcomings this over-view may have, being the result of unpreserved documentation from early stages, but also due to the “holes” in the memory of the past not so long ago.

## Figures and Tables

**FIGURE 3.**
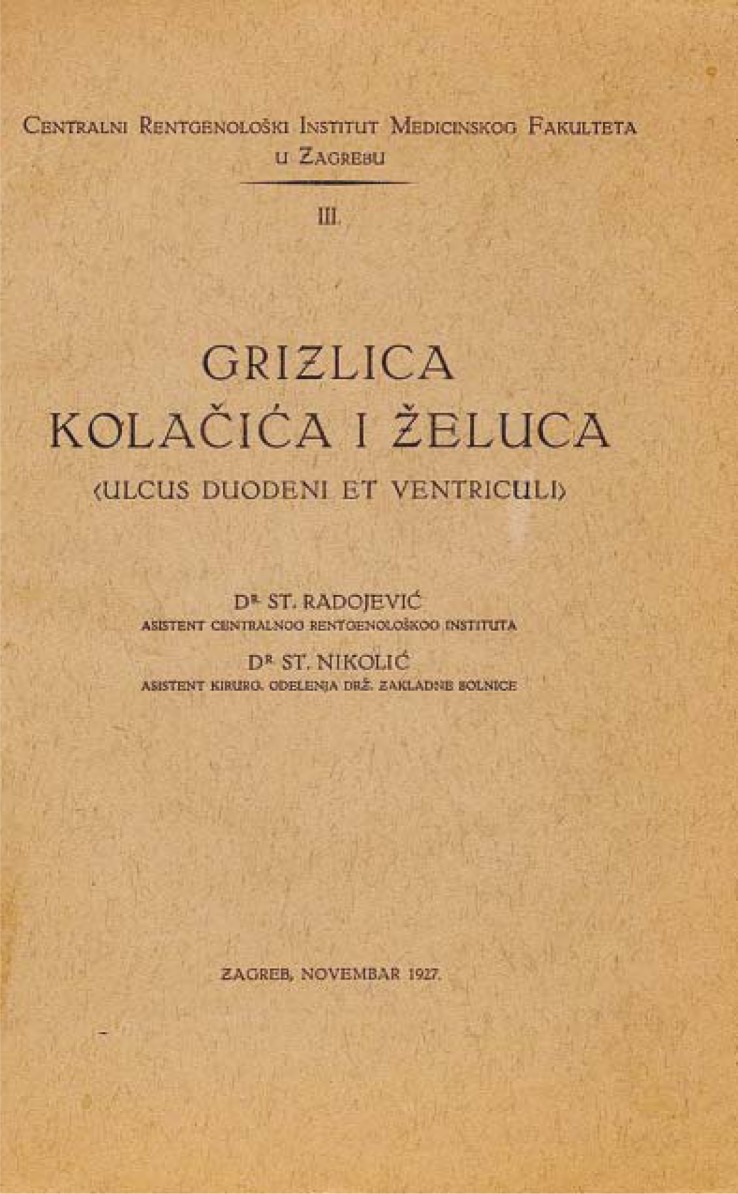
The book of Stevo Radojević and St. Nikolić. Radojević S, Nikolić S. *Grizlica kolačića i želuca [Ulcus duodeni* *et ventriculi].* Zagreb: Centralni rentgenološki institut Medicinskog fakulteta u Zagrebu [Central Roentgen Institute of Medical Faculty Zagreb]; 1927.

**FIGURE 4.**
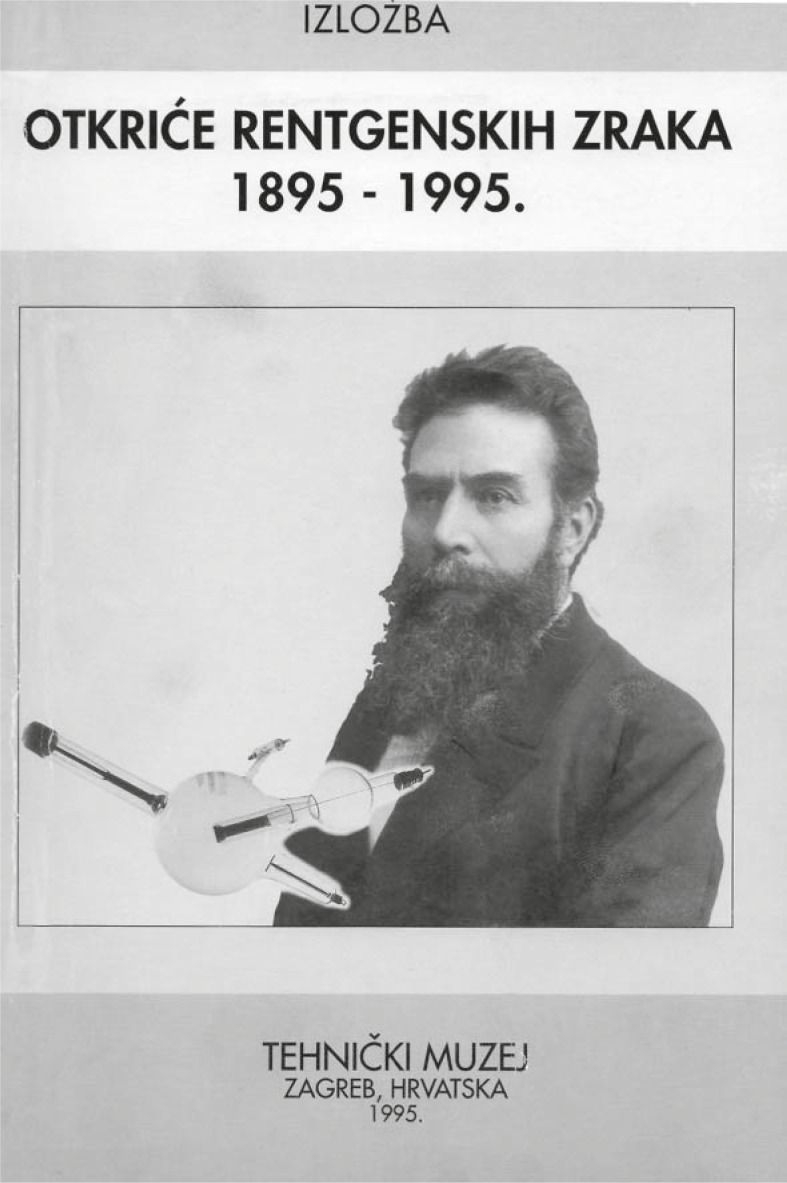
Izložba. Otkriće rentgenskih zraka 1895–1995“. [Exhibition, Discovery of x-Ray, 1895-1995]. Zagreb 14.12.1995 – 18.02.1996. Zagreb: Tehnički muzej Zagreb [Technical museum Zagreb]; 1995.

**TABLE 1.** Publications of radiology that were published in Croatia with help of Croatian radiology experts and teachers

Radojević S, Nikolić S. Grizlica kolačića i želuca [Ulcus duodeni et ventriculi]. Zagreb: Centralni rentgenološki institut Medicinskog fakulteta u Zagrebu [Central Roentgen Institute of Medical Faculty Zagreb]; 1927. (Figure 3)
Popović L, Smokvina M. Pregled naše rentgenološke literature. Zagreb: Centralni rentgenološki institut Medicinskog fakulteta u Zagrebu; 1927.
Smokvina M. Klinička rentgenologija, kosti i zglobovi [Clinical roentgenology, bones and joints]. Zagreb: Yugoslav Academy of Sciences and Arts; 1959. (Figure 4)
Hodges FJ, Lampe I, Floyd HF. Radiology for Medical Students. [Radiologija za studente medicine]. 4^th^ Edition. Chicago: Year Book Medical Publisher Inc.;1964; Zagreb:Školska knjige Zagreb; 1976
Petrovčić F. Leksikon radioloških pojmova. Zagreb: Leksikografski zavod M. Krleža Zagreb; 1977.
Okrugli stol o intervencijskoj radiologiji. Šimunić S, Gürtl R, editors. Zagreb: Zavod za radiologiju KBC Zagreb; 1981.^6^
Intervencijska radiologinja – perkutana transluminalna angioplastika renalnih, koronarnih i perifernih arterija.Šimunić S, Šesto M, editor. Zagreb: Sekcija za radiologiju, Kardiološka i nefrološka sekcija Zbora liječnika Hrvatske; 1985.^7^
Intervencijska radiologija. Mašković J, Boschi S, Stanić I, editors. Split; Sekcija za radiologiju Zbora liječnika Hrvatske – Podružnica Split; 1986.^8^
Plavšić B. Radiologija probavnog kanala. Zagreb: Školska knjiga Zagreb; 1986.
Petrovčić F. Opća radiologija. Zagreb: Sveučilišna naklada, Liber Zagreb; 1986.
Hebrang A, Petrovčić F. Radijacija i zaštita u medicinskoj dijagnostici. Zagreb, Beograd: Medicinska knjiga; 1987.
Bešenski N, Škegro N. Radiografska tehnika skeletal.Zagreb: Školska knjiga Zagreb; 1987.
Škarica R, Potočki K. Radiološki atlas reumatskih bolesti. Zagreb, Beograd: Medicinska knjiga; 1989.
Plavšić B. Radiologija probavnog kanala. 2^nd^ edition. Zagreb: Školska knjiga Zagreb; 1990.
Radiologija. Agbaba M, Lovrenčić M, editors. 1^st^ edition. Zagreb: Medicinska naklada Zagreb; 1994.
WHO Scientific Group on Clinical Diagnostic Imaging. [Izbor dijagnostike u kliničkoj praksi – Izbor radioloških dijagnostičkih postupaka]. Hebrang A, translator and editor. Zagreb: HZZO; 1996.
Frković M. Radiološki atlas probavnog sustava djece. Zagreb: Informator; 1998.
Strugačevac P. Teorijska osnova imaging CT tehnike. Osijek: KB Osijek; 1999.
Brkljačić B. Dopler krvnih žila. Zagreb: Medicinska naklada Zagreb; 2000.
Radiologija. Hebrang A, Lovrenčić M, editors. 2^nd^ edition. Zagreb: Medicinska naklada Zagreb; 2001.
Chapman S, Nakilney R. Pomoć u radiološkoj diferencijal-noj dijagnostici. [Translation from English]. Gotovac N, editor. Požega: Self-published; 2005.
Pichler E. Ultrazvučni atlas dojke-diferencijalna dijagnoza i intervencije. Zagreb: Školska knjiga Zagreb; 2005.
Pavić L, Radoš M. Mali medicinski leksikon magnetne rezonancije. Zagreb: Školska knjiga Zagreb; 2005.
Seminari iz kliničke radiologije. Janković S, editor. Split: MF Split; 20905.
Stojanović J. Trzajna ozljeda-riješena enigma. Zagreb: Sveučilišna tiskara; 2006.
Radiologija. Hebrang A, Klarić-Čustović R, editors. Zagreb: Medicinska naklada Zagreb; 2007.^9^,^10^
Miletić D. Skeletna radiografija. Rijeka: Glosa; 2008.
Odabrana poglavlja intervencijske radiologije. Mašković J, Janković S, editors. Split: MF Split; 2008.
Dentalna radiografija. Janković S, Miletić D, editors. Split: MF Split; 2009.
Brkljačić B. Vaskularni ultrazvuk. Zagreb: Medicinska naklada Zagreb; 2010.
